# Effects of heat stress on 16S rDNA, metagenome and metabolome in Holstein cows at different growth stages

**DOI:** 10.1038/s41597-022-01777-6

**Published:** 2022-10-22

**Authors:** Lei Feng, Yu Zhang, Wei Liu, Dewei Du, Wenbo Jiang, Zihua Wang, Zhonghua Wang, Zhiyong Hu

**Affiliations:** College of Animal Science and Technology, Shandong Agricultural University, Taian, China

**Keywords:** Metagenomics, Microbiome

## Abstract

Heat stress is an important issue in dairy cattle feeding management affecting summer health and economic efficiency. This experiment combined 16S rDNA sequencing(3,864,982 tags, 30 sequencing data), metagenomic sequencing(1,269,441,128 reads, 18 sequencing data), metabolomics analysis(72 sequencing data) and blood index analysis. Ten cows in each animal type (growing heifers, heifers, and lactating cows) were selected for sample collection in April and August. Here, we characterized both the changes in metabolites, rumen microbial communities and their functional potential and the effects of heat stress on serum biochemical, immune, oxidative stress, and hormonal indices derived from rumen fluid and serum samples from cows during different growth stages and in different climates. The generated data expand the resources for the rumen microbiome related to heat stress and age and provide useful datasets for research on developing therapeutic strategies to achieve high summer milk production in cows. These datasets will help researchers study the effects of heat stress on the physiological metabolism of Holstein cows and the time-dependent changes associated with growth stages.

## Abstract (summary)

**Table:** 

Measurement(s)	Metabolite abundances in serum • Metabolite abundances in rumen fluid • taxonomic diversity assessment by targeted gene surveyin rumen fluid • metagenome in rumen fluid
Technology Type(s)	Liquid Chromatography Mass Spectrometry • next generation DNA sequencing • Next Generation Sequencing
Factor Type(s)	heat stress and growth stages
Sample Characteristic - Organism	Bos taurus
Sample Characteristic - Environment	growing heifers, heifers, and lactating cows were selected in April and August of Shandong Province

## Background & Summary

As the scale of Holstein dairy cow breeding continues to increase, the feeding environment has been improved to a certain extent, but the continuing rise of annual average temperature presents a more serious challenge to larger-scale dairy farming^1,2^. Cows are extremely sensitive to hot environments^3^. In Shandong Province from July to August, relatively mild chronic heat stress^4^ caused by the increased temperatures in summer is the major cause of stress in dairy cows. Anaerobic fermentation in the rumen, exercise and milk production lead to metabolic heat producing, can cause heat stress in cows when metabolic heat production exceeds their potential to dissipate heat. In particular, the high temperature and humidity in summer restrict the heat dissipation from the body surface of cows and continuous increase the heat in metabolic production^5^, which can intensify the occurrence of heat stress in cows.

Previous studies have shown that once heat stress occurs in cows, the hypothalamic-pituitary-adrenal (HPA) axis^6^ and the regulation of the pro-adrenocortical hormone nervous system can be activated, which not only negatively affects the growth performance and blood biochemical parameters, but also interferes with the metabolic and physiological processes required for optimal productivity as well as immunity and negatively affects the disease resistance. A study conducted by Guo *et al*.^7^ reported that heat stress leads to disturbance of redox balance and increase of free radical concentration in the body, which suppresses the antioxidant defense system of cows and leads to oxidative stress. In addition, cows with oxidative stress have increased peroxides, damaged membrane systems and reduced immunity^8^, exacerbated the level of heat stress and may be associated with the development of multiple diseases.

Heat stress is one of the main stresses affecting the production of dairy cows and it has caused massive losses to animal husbandry production and economic development^8,9^. It is important to explore the effects of heat stress on Holstein dairy cows. As ruminants, the digestion of nutrients by cows is closely related to the rumen^10,11^. The rich microorganisms contained in the rumen are an indispensable link in the digestion of animal nutrients and play a key role in the conversion of plants into milk by dairy cows^12^. Numerous studies have shown that the interactions between rumen microorganisms and the host affect host metabolic processes^13,14^. The connection between rumen microbial community composition and change and host production performance is an important breakthrough for heat stress affecting dairy farming technology.

Further exploration of host-microbiota interactions will require sufficient datasets to facilitate the identification of beneficial microorganisms in the rumen microbiota for cow production during heat stress. Currently, most of the studies on the effects of heat stress on Holstein cows are now based on amplicon sequencing of various microbial marker genes and are specific to a particular stage of Holstein cows; however, fewer studies have been conducted on the long-term effects of Holstein cows exposed to summer heat stress for their physiology. Therefore, this study was conducted on Holstein cows at three stages, growing heifers, heifers and lactating cows, combining 16S rDNA sequencing, metagenomics and metabolomics to more precisely explore the effects of heat stress on microbial composition, function and metabolism in Holstein cows.

We described the datasets of 16S rDNA sequencing, metagenomic sequencing, metabolomics analysis of rumen fluid samples and metabolomics analysis of serum samples from Holstein cows at different growth stages, as well as two temperature and humidity index conditions, namely, normal and heat stress states. A total of 3,864,982 ± 5,330 raw tags were generated from 30 samples for 16S rDNA sequencing, and 1,269,441,128 ± 3,415,214 raw reads were generated from 18 samples for shotgun sequencing. A total of 36 rumen fluid and 36 blood samples (Table 1) were analyzed on the same instrument in this study, yielding a metabolic dataset for normal and heat-stressed Holstein cows at different stages of growth. The data provided allow not only an in-depth analysis of the composition and function of the microbiome of Holstein cows but also a comparison of different growth stages to explore time-dependent changes in rumen microbes and metabolism. The raw and processed data are available to the research community and may be useful in diagnosing cattle suffering from current or future heat stress.

**Table 1:** Sample information in this study.

Library	Experiment	Condition	Sample	Material
16S rDNA	Normal	Growing Heifers	n = 5	Rumen Fluid
		Heifers	n = 5	
		Lactating Cows	n = 5	
	Heat Stress	Growing Heifers	n = 5	
		Heifers	n = 5	
		Lactating Cows	n = 5	
Metagenome	Normal	Growing Heifers	n = 3	
		Heifers	n = 3	
		Lactating Cows	n = 3	
	Heat Stress	Growing Heifers	n = 3	
		Heifers	n = 3	
		Lactating Cows	n = 3	
Metabolome	Normal	Growing Heifers	n = 6	
		Heifers	n = 6	
		Lactating Cows	n = 6	
	Heat Stress	Growing Heifers	n = 6	
		Heifers	n = 6	
		Lactating Cows	n = 6	
Metabolome	Normal	Growing Heifers	n = 6	Blood
		Heifers	n = 6	
		Lactating Cows	n = 6	
	Heat Stress	Growing Heifers	n = 6	
		Heifers	n = 6	
		Lactating Cows	n = 6	

## Methods

### Ethical statement

All experimental animals involved in the present study were housed according to the principles of the Institutional Animal Care and Use Committee of Shandong Agricultural University.

### Study design

The experiment was divided into 2 phases (heat stress and normal periods) and Holstein cows at 3 growth stages (growing heifers, heifers, and lactating cows) were selected to be sampled during the heat stress and normal period, respectively. According to growth stage and temperature humidity index (THI), the animals were differently treated whereby: normal group (C-N, H-N, D-N, THI mean = 50.76, n = 10) under natural conditions on April 7, 2021 and heat stress group (C-HS, H-HS, Dc-HS, THI mean = 81.11, n = 10) on August 6, 2021. The cows showed heat stress and normalcy more than three weeks before sampling, respectively.

### Management of holstein cows

The experiment was conducted at the Shandong Gaosu Dairy Farm (Jinan, China). Ten healthy growing heifers (6.93 ± 0.14 months of age), heifers (16.54 ± 0.11 months of age) and lactating cows with 2 litters (41.02 ± 0.36 months of age) of similar body condition were separately selected for this study.

### Sample collection and management

The THI was calculated as follows^15^:

$${\rm{THI}}=0.8\times {\rm{ambient}}\;{\rm{temperature}}+\frac{ \% {\rm{relative}}\;{\rm{humidity}}}{100}\times \left({\rm{ambient}}\;{\rm{temperature}}-14.4\right)+46.4$$

THI-related data were recorded three times per day (06:00, 14:00, and 22:00) and the average of the 3 measurements was taken as the THI of each day. The THIs of the normal and heat-stressed groups are as shown in Tables 2 and 3, respectively. Furthermore, rectal temperature and respiratory rate were measured 3 times per day (06:00, 14:00, and 22:00). Rectal temperature was measured using a veterinary thermometer and respiratory rate was determined by counting the number of thoracic rumbles in cows in 1 minute. The rectal temperature and respiratory rate of the normal and heat-stressed groups are as shown in Tables 4. The rumen fluid samples were collected through oral stomach tubes and obtained by squeezing and filtering through 3 layers of gauze. The rumen fluid samples were divided into lyophilized tubes and stored at −80 °C for further characterization of microbial composition and function, and metabolites.

**Table 2:** Average temperature and humidity and average temperature and humidity index in April.

Time	Average Temperature (°C)	Average Relative Humidity (%)	Average Temperature and Humidity Index(THI)
6:00	8.84	39.03	51.25
14:00	17.28	37.30	61.35
22:00	8.49	40.33	50.76

**Table 3:** Average temperature and humidity and average temperature and humidity index in August.

Time	Average Temperature (°C)	Average Relative Humidity (%)	Average Temperature and Humidity Index(THI)
6:00	26.50	79.04	77.15
14:00	34.90	67.66	88.28
22:00	27.10	78.11	77.94

The blood(10 mL) was collected from the caudal root vein of the test cow and placed in a procoagulant tube. The blood was centrifuged at 3,500 r/min for 10 min after 30 min at room temperature, and the supernatant was divided into 1.5 mL centrifuge tubes. To avoid variation, the separated serum samples were immediately stored in a liquid nitrogen tank and brought back to the laboratory to be stored in a refrigerator at −80 °C with appropriate labels (ID, sample type, and collection date) for blood markers and metabolomics.

**Table 4:** Rectal temperature and respiratory rate of Holstein cows at different stages.

Items	Groups						SEM
	C-N	C-HS	H-N	H-HS	D-N	D-HS	
Rectal TemperaTure /°C	38.82	39.16	38.55	38.90	38.59	38.92	0.08
Respiratory Rate /(bpm)	22.60	58.50	24.90	56.80	26.90	58.60	0.57

### Measurement of serum biochemical parameters, hormones, oxidative stress and immune indicators

The activities of serum alkaline phosphatase (ALP), aspartate aminotransferase (AST), alanine aminotransferase (ALT), glucose (GLU), triglycerides (TG), total cholesterol (TC), total protein (TP), albumin (ALB), and urea in serum were measured using the commercial kits of Nanjing Aoqing Biotech Co. (Nanjing, Jiangsu, China) by a full automation biochemistry analyzer (Beckman Coulter Model: AU680). In our study, Malondialdehyde (MDA) kit, the superoxide dismutase (SOD) kit, Glutathione peroxidase (GSH-PX) kit and total antioxidant capacity (T-AOC) kit were used to measure MDA, SOD, GSH-PX and T-AOC, respectively. The kits were purchased from Angle Gene, and the procedures of the assays were performed strictly according to the instructions provided in the kits. Determination of insulin (INS), prolactin (PRL), glucocorticoids (GC), triiodothyronine (T3), thyroxine (T4), cortisol (COR), interleukin-1β (IL-1β), interleukin-6 (IL-6), interleukin-2 (IL-2), interleukin-4 (IL-4), interleukin-10 (IL-10), tumor necrosis factor alpha (TNF-α), interferon-gamma (IFN-γ), immunoglobulin A (IgA), immunoglobulin G (IgG), immunoglobulin M (IgM), and heat shock protein 70 (HSP70) in serum samples using enzyme-linked immunosorbent assay (ELISA) kits according to instructions provided by the manufacturer (Angle Gene Ltd.). The detection results of serum biochemical parameters, hormones, oxidative stress and immune indicators are as shown in Tables 5, 6, 7 and 8.

**Table 5:** Effects of heat stress on biochemical indicators of blood in Holstein cows at different stages.

Items	Groups						SEM
	C-N	C-HS	H-N	H-HS	Dc-N	Dc-HS	
ALP, U/L	278.87	201.80	201.26	131.14	76.03	46.08	13.51
GLU, mmol/L	4.08	5.00	3.87	3.80	3.16	3.45	0.08
TC,mmol/L	2.83	3.35	2.95	2.76	5.86	6.29	0.20
TG,mmol/L	0.32	0.40	0.29	0.32	0.25	0.24	0.02
ALT,U/L	38.78	40.22	28.20	28.33	38.70	40.50	2.2
AST,U/L	70.30	66.51	64.58	65.54	83.27	94.85	3.32
TP,g/L	67.40	75.75	69.81	71.38	78.93	89.28	1.35
ALB,g/L	22.51	25.71	24.22	25.26	24.26	26.55	0.58
UREA,mmol/L	3.98	2.46	4.91	4.83	4.30	5.25	0.20

**Table 6:** Effects of heat stress on serum hormones at different stages in Holstein cows.

Items	Groups						SEM
	C-N	C-HS	H-N	H-HS	Dc-N	Dc-HS	
PRL, μIU/mL	3076.49	2707.12	3192.27	2877.77	2892.63	2560.43	53.1
GC, ng/mL	33.05	24.65	37.36	29.02	29.15	21.26	0.28
T_3_, ng/mL	1.34	1.76	1.04	1.35	1.78	2.29	0.02
T_4_, ng/mL	68.46	87.17	56.93	76.09	81.71	99.18	1.06
INS, mU/mL	13.31	8.91	14.87	11.15	11.69	7.25	0.15
COR, ng/mL	102.66	73.34	117.58	97.19	87.62	57.17	1.51
Degree of INS reduction*	0.33		0.25		0.38		0.01

Notes: *The calculation formula is (N-S)/N

**Table 7:** Effects of heat stress on serum immune indicators in Holstein cows.

Items	Groups						SEM
	C-N	C-HS	H-N	H-HS	Dc-N	Dc-HS	
IL-1β, pg/mL	154.82	183.36	134.14	164.16	174.86	202.30	2.95
IL-6, pg/mL	221.10	250.27	204.89	233.15	238.88	265.38	2.33
IL-2, pg/mL	16.05	22.07	14.08	20.02	18.08	24.01	0.19
IL-4, pg/mL	455.22	416.38	475.17	433.17	436.10	394.62	6.31
IL-10, pg/mL	407.78	363.02	428.70	382.88	385.75	344.63	5.83
TNF-α, ng/L	128.27	155.51	115.36	141.05	140.40	165.00	2.45
IFN-γ, pg/mL	540.59	699.57	492.92	662.53	601.00	760.41	11.5
IgA, μg/mL	27.57	19.04	30.53	22.14	24.50	16.19	0.26
IgG, μg/mL	262.03	235.66	271.51	245.54	248.34	222.17	3.92
IgM, μg/mL	21.97	17.18	24.06	19.04	20.02	15.08	0.34
HSP70, ng/mL	10.34	8.27	11.68	9.43	9.05	6.86	0.13

**Table 8:** Effects of heat stress on serum oxidative stress indicators in Holstein cows at different stages.

Items	Groups						SEM
	C-N	C-HS	H-N	H-HS	Dc-N	Dc-HS	
MDA,nmol/mL	4.41	5.86	3.51	5.03	5.73	7.18	0.09
SOD,U/mL	80.43	69.40	85.01	74.29	75.82	64.83	0.57
T-AOC,mmol/L	0.23	0.15	0.27	0.20	0.20	0.12	0.002
GSH-PX,U/mL	14.38	12.16	15.04	12.76	13.46	11.23	0.27

### 16S rDNA and metagenomic sequencing

Total DNA of the samples was extracted using HiPure Stool DNA Kits (Magen, Guangzhou, China). The concentration of the purified DNA was measured for 16S rDNA and metagenomic sequencing using a NanoDrop spectrophotometer (Thermo Fisher Scientific, Massachusetts, USA). The DNA purity was assessed with gel electrophoresis, and a gel imaging system. The DNA quality was detected using a Qubit (Thermo Fisher Scientific, Waltham, MA) and NanoDrop (Thermo Fisher Scientific, Waltham, MA) accordingly.

Using V3-V4 hypervariable region universal primers (341 F:CCTACGGGNGGCWGCAG; 806 R: GGACTACHVGGGTATCTAAT), amplified the V3-V4 region-specific region to obtain the target PCR fragment. Amplicons extracted from 2% agarose gels were purified using an AxyPrep DNA Gel Extraction Kit (Axygen Biosciences, Union City, CA, USA) and quantified using an ABI StepOnePlus Real-Time PCR System (Life Technologies, Foster City, USA) according to the manufacturer’s instructions. Targeted 16S sequencing libraries were prepared according to the 16S Metagenomics Sequencing Library Preparation protocol (Illumina, San Diego, CA, USA) in combination with sequencing primers and sequencing adapters. Sample with a bright band between 430–470 bp was used for library construction. The concentration of 16S rDNA libraries was assessed using an Agilent 2100 bioanalyzer instrument (Agilent DNA 1000 Reagents) and a Genomic DNA Sample Prep Kit for the Illumina Nova6000 Platform. Purified amplicons were pooled in equimolar amounts and sequenced (PE250, 2 × 250 paired-end mode) on an Illumina platform according to standard protocols of the NovaSeq. 6000 sequencing platform (Illumina, San Diego, CA, USA) using the pair-end technology and single-ended indexes by Guangzhou Genedenovo Biotechnology Co., Ltd (Guangzhou, China), as recommended by the manufacturer.

Sample with a bright band between 300–400 bp was used for library construction. Metagenome libraries were constructed using a Genomic DNA Sample Prep Kit (Illumina, San Diego, CA, USA) following the manufacturer’s recommendations. The concentration of metagenome libraries was assessed using an Agilent 2100 Bioanalyzer instrument (Agilent DNA 1000 Reagents) and a Genomic DNA Sample Prep Kit for Illumina NovaSeq. 6000 Platform. PCR products were purified using an AMPure XP system (Beckman Coulter, Brea, CA, USA), and metagenomic sequencing libraries were checked for appropriate size distribution by a 2100 Bioanalyzer (Agilent, Santa Clara, CA, USA) and quantified using real-time PCR. Metagenomic sequencing was performed on an Illumina NovaSeq. 6000 sequencer using paired-end technology (PE150, 2 × 150 paired-end mode) by Guangzhou Genedenovo Biotechnology Co., Ltd (Guangzhou, China), as recommended by the manufacturer.

### LC-MS analysis

We carried out untargeted LC-MS profiling analysis of samples. The serum (100 μL) was placed in EP tubes and resuspended in 400 μL of prechilled 80% methanol by thoroughly vortexing. Then, the samples were incubated on ice for 5 min and centrifuged at 15,000 × g and 4 °C for 20 min. Some of the supernatant was diluted to a final concentration containing 53% methanol with LC-MS-grade water. The samples were subsequently transferred to a fresh Eppendorf tube and then centrifuged at 15,000 × g and 4 °C for 20 min. Finally, the supernatant was injected into an LC-MS/MS system for analysis using a Hypesil Gold aQ^16,17^. The column temperature was 40 °C, and the flow rate was 0.2 mL/min. In positive mode, the mobile phase of composition A consists of 0.1% formic acid, and B was methanol. In negative mode, the mobile phase of composition A was 5 mM ammonium acetate and pH = 9.0, and B was methanol.

The instrument and LC-MS setup were as follows: a Thermo Scientific Q ExactiveTM HF-X with a dual sprayer ESI source attached to a Vanquish UHPLC (Thermo Fisher, Germany) comprised of a vacuum degasser, binary pump, thermostated autosampler and column oven. Using Thermo Scientific UltiMate WPS-3000 Autosampler and chromatography instrument: Thermo Scientific Vanquish UHPLC System.

### Microbial community profiling

Raw reads were further filtered and trimmed to remove low-quality bases and adaptor sequences according to the following rules using FASTP^18^ (version 0.18.0):

1. Reads containing more than 10% unknown nucleotides (N) were removed;
2. Reads containing less than 50% bases with quality (Q-value) > 20 were removed.

The tag was obtained by splicing the double-ended reads using FLASH (version 1.2.11)^19^, and then filtering the tag to obtain the data called Clean tag. For raw tag filtering, we used specific filtering conditions (the default quality threshold is ≤3; the default length is 3 bp) to obtain the high-quality clean tags. Next, the clustering was performed based on the clean tag, the clean tags were clustered into operational taxonomic units (OTUs) based on ≥97% similarity using the UPARSE^20^ (version 9.2.64) pipeline. All chimeric tags detected during the clustering comparison process were removed by the UCHIME algorithm of USEARCH^21^, and the data finally obtained were effective tags for further analysis. Species annotation via RDP classifier (version 2.2)^22^ based on Bayesian algorithm. Sequence alignment was performed using Muscle^23^ (version 3.8.31), and a phylogenetic tree was constructed using FastTree^24^ (version 2.1). Then, weighted and unweighted UniFrac distance matrices were generated by the GuniFrac package^25^ (version 1.0).

### Microbial function profiling

Raw data from the Illumina platform were filtered using FASTP (version 0.18.0) by the following standards to obtain high-quality clean reads^18^: (1) removing reads with ≥10% unidentified nucleotides (N); (2) removing reads with ≥50% bases having Phred quality scores ≤20; and 3) removing reads aligned to the barcode adapter. After filtering, the resulting clean reads were used for genome assembly by FLASH (version 1.2.11). The predicted genes ≥300 bp in length from all samples were pooled and combined based on ≥95% identity and 90% read coverage using CD-HIT (version 4.6)^26^ to reduce the number of redundant genes for the downstream assembly step. High-quality reads of each sample were compared to the overall nonredundant gene set using Bowtie (version 2.2.5)^27^ to calculate the abundance of each gene in each sample. The final gene catalog was obtained from nonredundant genes with a gene read count >2. Nonredundant gene sets were aligned to the functional database using DIAMOND (version 0.9.24) to obtain the specific function of each gene. These functional databases include the National Center for Biotechnology Information (NCBI) nonredundant protein (Nr) database, Kyoto Encyclopedia of Genes and Genomes (KEGG), and Evolutionary Genealogy Of Genes: Nonsupervised Orthologous Groups (eggNOG). Clean reads were used to generate taxonomic profiles using Kaiju (version 1.6.3)^28^.

### Data processing and metabolite identification

The raw data files generated by UHPLC-MS/MS were processed using Compound Discoverer 3.1 to perform peak alignment, peak picking, and quantitation for each metabolite. The main parameters were set as follows: retention time tolerance, 0.2 minutes; actual mass tolerance, 5 ppm; signal intensity tolerance, 30%; signal/noise ratio, 3; and minimum intensity, 100,000. After that, peak intensities were normalized to the total spectral intensity. The normalized data were used to predict the molecular formula based on additive ions, molecular ion peaks and fragment ions. Then, peaks were matched with the mzCloud (https://www.mzcloud.org/) and mz vault and mass list databases to obtain accurate qualitative and relative quantitative results. Statistical analyses were performed using the statistical software R (R version R-3.4.3), Python (Python 2.7.6 version) and CentOS (CentOS release 6.6).

## Data Records

The raw reads files (fastq format) for each sample of 16S rDNA and metagenomic sequencing have been uploaded to the NCBI Sequence Read Archive (SRA) under project number PRJNA850536^29^ and PRJNA850514^30^. The raw LC-MS data files of rumen fluid samples and serum samples were converted to.mgf format by using ProteoWizard package (http://proteowizard.sourceforge.net), respectively were deposited and are publicly available at the MetaboLights database^31^ (http://www.ebi.ac.uk/metabolights) of the European Bioinformatics Institute under MTBLS5132^32^ and MTBLS5148^33^. All data can be used without restrictions.

## Technical Validation

The DNA quality of the metagenomics was determined, and the DNA total amount ≥1 μg, concentration ≥30 ng/μL indicated that the DNA quality was qualified. The concentration of metagenome libraries was assessed using an Agilent 2100 Bioanalyzer instrument (Agilent DNA 1000 Reagents) and a Genomic DNA Sample Prep Kit for Illumina NovaSeq. 6000 Platform, the libraries with qualified concentration (≥10 nM) and volume (15 μL–100 μL) were subjected for sequencing.

**Fig. 1:**
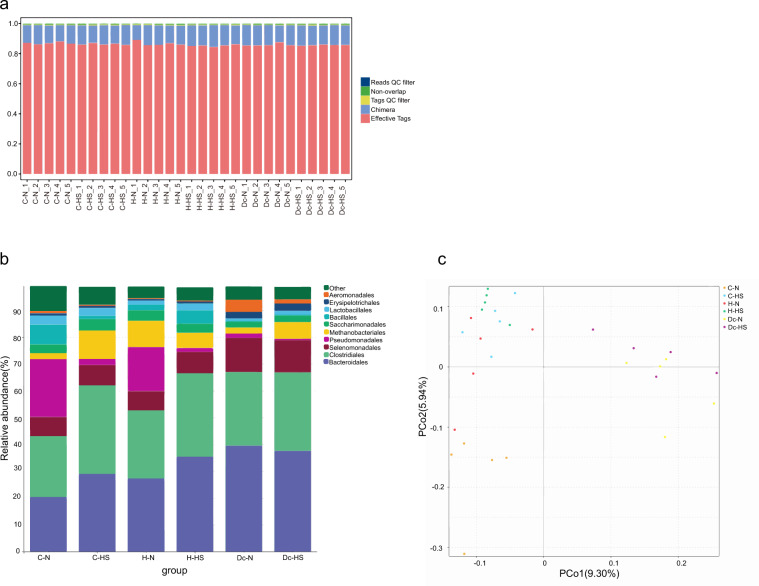
The quality assessment of 16S rDNA sequencing data. (**a**) Bar graph of sequencing data volume and filtering analysis effects. Reads QC filter: low-quality reads; Non-overlap: unassembled reads without overlap; Tag QC filter: tags that do not pass the “tag filter”; Chimera: number of chimera tags. (**b**) Bacterial community composition at the order level in this study. Taxonomic assignments of the 10 most abundant taxa are given. (**c**) Principal Coordinate Analysis (PCoA) of all datasets based on beta-diversity with unweighted-unifrac.

**Fig. 2:**
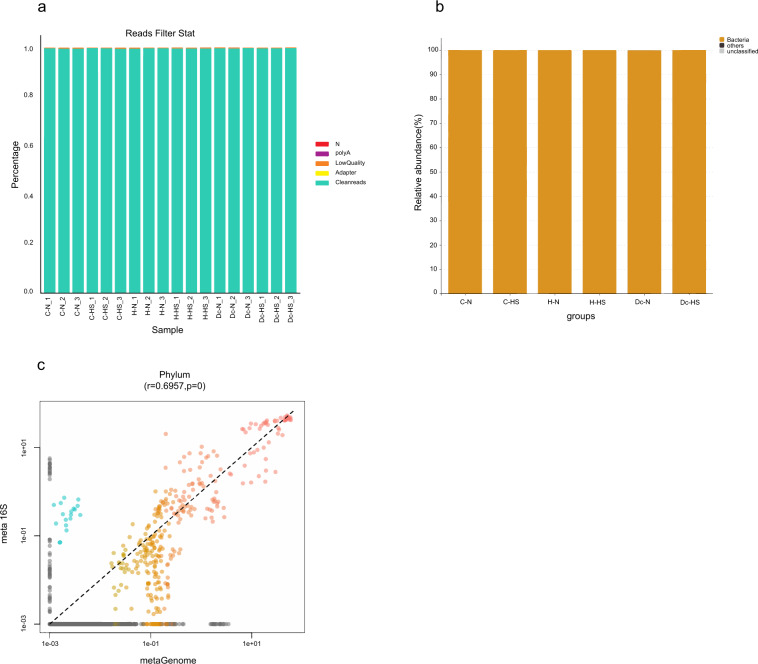
The quality assessment of metagenomic sequencing data. (**a**) Data preprocessing distribution map. Adapter: reads with adapters; Low Quality: low-quality reads; PolyA: number of reads containing polyA (%); N: single-ended reads contain more than 10% of N bases. (**b**) The taxonomic prediction of raw reads is shown at the domain level. The “others” shown here means reads that contain the virus, archaea, unclassifed taxa, and other sequences. (**c**) Correlation of taxonomic compositions between 16S rDNA sequencing and metagenomic sequencing. “R” and “P” indicate the Pearson’s R and signifcance of the pairing, respectively.

Quality control of 16S rDNA sequencing reads was performed using FastP (version 0.18.0) and FLASH (version 1.2.11). Filtering tags parameters refer to previous studies^34^. The quality assessment of 16S rDNA sequencing is shown in Fig. 1a. The orders Bacteroidales, Clostridiales, Selenomonadales, Pseudomonadales, and Methanobacteriales dominated the bacterial communities (Fig. 1b). All the metagenomic sequencing samples underwent data quality control to remove contaminants and trim reads with a low quality below 0.25%. The metagenomic clean reads were evaluated for their read qualities. The observed quality distribution showed that clean reads accounted for more than 99%, indicating no quality problems (Fig. 2a). The relative abundance of bacteria was 100% (Fig. 2b). Bacterial taxonomy comparison between 16S rDNA sequencing and metagenomic sequencing data demonstrated the strong consistency of the bacterial microbiome structure (Fig. 2c).

**Fig. 3:**
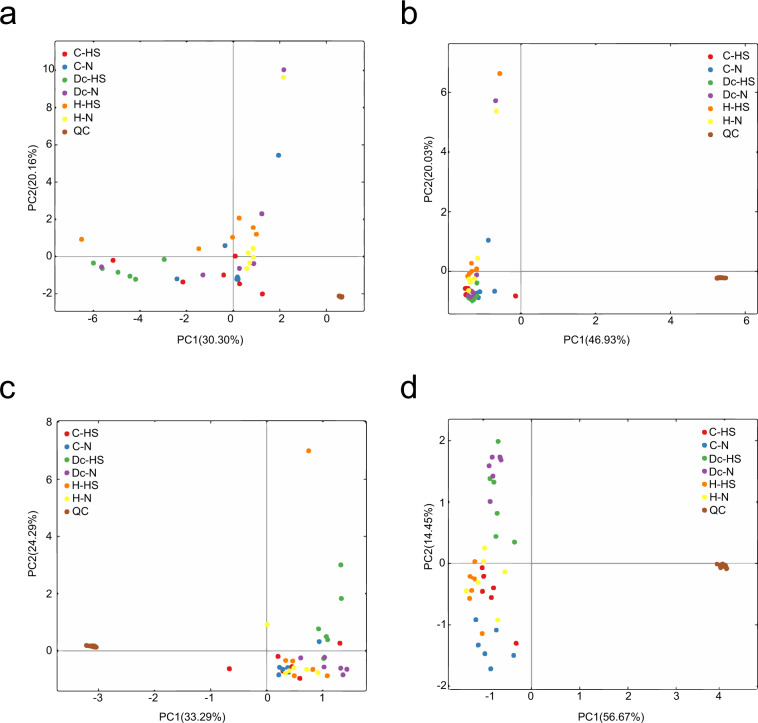
The principle component analysis(PCA) of quality control. (**a**) Ruminal fluid samples in positive mode; (**b**) Ruminal fluid samples in negative mode; (**c**) Blood samples in positive mode; (**d**) Blood samples in negative mode.

To ensure unbiased data production, randomization orders were created and followed for sample extraction, and MS run orders. All samples were analysed within the same batch. Samples were randomized based on heat stress and growth stage. QC samples were used for quality control in our study of metabolomics is based on mass spectrometry^35^. Unsupervised principal component analysis (PCA) was applied to find the main source of variation within the data, to check population homogeneity (Fig. 3), the results of the QC samples were clustered distinctively, suggesting that the test data were reliable.

## Data Availability

Serum enzyme activity, antioxidant capacity, and immune parameter were statistically processed and analyzed using R version 4.0.3, default parameters or parameters recommended by the developer were used.
